# Circadian Clock-Controlled Checkpoints in the Pathogenesis of Complex Disease

**DOI:** 10.3389/fgene.2021.721231

**Published:** 2021-09-07

**Authors:** Min-Dian Li, Haoran Xin, Yinglin Yuan, Xinqing Yang, Hongli Li, Dingyuan Tian, Hua Zhang, Zhihui Zhang, Ting-Li Han, Qing Chen, Guangyou Duan, Dapeng Ju, Ka Chen, Fang Deng, Wenyan He, Biological Rhythm Academic Consortium in Chongqing (BRACQ)

**Affiliations:** ^1^Department of Cardiology and the Center for Circadian Metabolism and Cardiovascular Disease, Southwest Hospital, Third Military Medical University (Army Medical University), Chongqing, China; ^2^Medical Center of Hematology, The Xinqiao Hospital of Army Medical University, Chongqing, China; ^3^Department of Anesthesiology, The Second Affiliated Hospital, Chongqing Medical University, Chongqing, China; ^4^Department of Obstetrics and Gynaecology, The First Affiliated Hospital of Chongqing Medical University, Chongqing, China; ^5^Department of Obstetrics and Gynaecology, The Second Affiliated Hospital of Chongqing Medical University, Chongqing, China; ^6^Key Lab of Medical Protection for Electromagnetic Radiation, Ministry of Education of China, Institute of Toxicology, College of Preventive Medicine, Army Medical University (Third Military Medical University), Chongqing, China; ^7^Research Center for Nutrition and Food Safety, Institute of Military Preventive Medicine, Army Medical University, Chongqing, China; ^8^Key Laboratory of Extreme Environmental Medicine, Department of Pathophysiology, College of High Altitude Military Medicine, Ministry of Education of China, Army Medical University (Third Military Medical University), Chongqing, China; ^9^Key Laboratory of High Altitude Medicine, PLA, Army Medical University (Third Military Medical University), Chongqing, China; ^10^Department of Dermatology, The First Affiliated Hospital of Chongqing Medical University, Chongqing, China

**Keywords:** circadian clock, complex disease, checkpoint, Nr1d1/Rev-erbα, melatonin, circadian rhythm, diabetes complications, systems biology

## Abstract

The circadian clock coordinates physiology, metabolism, and behavior with the 24-h cycles of environmental light. Fundamental mechanisms of how the circadian clock regulates organ physiology and metabolism have been elucidated at a rapid speed in the past two decades. Here we review circadian networks in more than six organ systems associated with complex disease, which cluster around metabolic disorders, and seek to propose critical regulatory molecules controlled by the circadian clock (named clock-controlled checkpoints) in the pathogenesis of complex disease. These include clock-controlled checkpoints such as circadian nuclear receptors in liver and muscle tissues, chemokines and adhesion molecules in the vasculature. Although the progress is encouraging, many gaps in the mechanisms remain unaddressed. Future studies should focus on devising time-dependent strategies for drug delivery and engagement in well-characterized organs such as the liver, and elucidating fundamental circadian biology in so far less characterized organ systems, including the heart, blood, peripheral neurons, and reproductive systems.

## Introduction

To align with the daily cycling of environmental conditions associated with the Earth’s rotation, terranean and subterranean organisms have evolved an internal circadian (Latin words: circa, about; dies, day) clock system to organize metabolism, physiology, and behavior (Bass and Lazar, 2016; Zhang et al., 2020b; López-Otín and Kroemer, 2021). The circadian clock manifests as internal and resettable (known as entrainable in the field) circadian rhythms at the molecular, physiological, and organismal levels. The circadian clock system in mammals is organized in a hierarchical manner, orchestrated by a master pacemaker in the hypothalamus. The circadian clock in individual cells is genetically similar and composed of transcriptional-(post)translational feedback loops.

In a time when fundamental and preclinical research on circadian rhythms is progressing at an unprecedented speed, the concept of time medicine (or chronomedicine, circadian medicine) is emerging as a new dimension in medicine (Cederroth et al., 2019; Panda, 2019; Allada and Bass, 2021). Here, we focused on research in the role of the circadian clock in major organs/tissues related to the pathogenesis of complex diseases, particularly those with metabolic disorders, and summarized tissue-specific circadian clock-controlled checkpoints as genes, proteins, and biological pathways that would be valuable for time medicine.

### The Architecture of the Circadian System in Mammals

**FIGURE 1:**
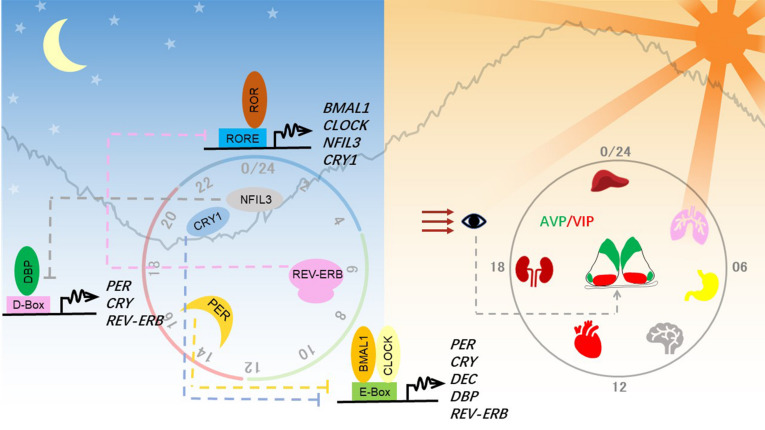
Molecular and anatomical structure of the circadian clock system. **Left panel:** Molecular mechanisms of the circadian clock mainly consist of three interlocked transcriptional-translational feedback loops. Transcriptional factors activate the transcription of repressor genes via binding to promoter regions containing different combinations of three *cis*-elements and thereby generating a different phase. These protein products of the repressor genes then enter the nucleus and feed back to repress their own transcription to close the circadian loop. **Right panel:** Circadian clocks exist in almost all tissues in mammals. The hypothalamic suprachiasmatic nucleus (SCN) in the brain serves as the master clock or the central clock, which is entrained by light through the retinohypothalamic tract pathway. Neuropeptides AVP and VIP maintain the robust cell-autonomous rhythmicity and light entrainment, respectively, in the SCN. The SCN clock synchronizes slave clocks in peripheral tissues through neuronal and humoral pathways throughout the body.

The architecture of the mammalian circadian clock system is established by a pacemaker located in the suprachiasmatic nucleus (SCN) of the hypothalamus (Allada and Bass, 2021). Light activates the non-cone non-rod melanopsin 4-expressing retinal ganglion neurons in the retina, transmitting signals to the SCN through a retinohypothalamic tract. The phase of the SCN is readily entrained by light, but seems refractory to other time signals associated with feeding and body temperature (Buhr et al., 2010; Reinke and Asher, 2019). The SCN coordinates clocks in non-SCN cells and tissues via neuronal or humoral connections and metabolic signaling associated with feeding rhythm. Feeding rhythm is crucial in clock synchronization in the body (Reinke and Asher, 2019), yet different peripheral clocks seem to respond with a broad spectrum of kinetics (Xin et al., 2021). Inverted feeding readily reverses circadian rhythms in liver and adipose tissue (Manella et al., 2021; Xin et al., 2021). Recently, a multi-omics study indicated that diurnal rhythms of transcriptomes, metabolomes, and clocks in the heart and kidney are resistant to circadian entrainment by feeding rhythm (Xin et al., 2021). The tissue-specific response to inverted feeding is gated by the temporal signaling from the SCN clock and the liver-clock (Manella et al., 2021; Xin et al., 2021). Thus, the coordinated actions of the SCN-peripheral clock axes orchestrate the harmony of mammalian physiological activities throughout the day (Figure 1), including sleep-wake cycle, endocrine secretion, blood pressure, energy metabolism, and urine production (Bass and Lazar, 2016).

Clocks in individual neurons of the SCN pacemaker are coupled to establish a robust circadian rhythm, which can last for weeks *in vitro* without dampening (Welsh et al., 2009). This strong intercellular coupling property is crucial for organizing the body’s clock. Both gap junctions and paracrine neurotransmitter signaling contribute to intercellular coupling. The SCN pacemaker is made of a pair of neuronal lobes containing ∼10,000 neurons, which are divided into a ventral core region and a dorsal shell region. The core region sits above the retinohypothalamic tract and receives input signals from environmental light. The shell region receives signals from the core region through neuronal projections and gap junctions. These two regions can be distinguished by the concentration of different neuropeptides, represented by vasoactive intestinal polypeptide (VIP) in the core neurons and arginine vasopressin (AVP) in the shell neurons. Upon light entrainment, VIP is released from the core neurons, depolarizing shell regions, leading to the resetting of the SCN clock. Recently, Shan et al. (2020) devised a color-switch dual-color imaging system in mice and showed that AVP-expressing shell neurons are crucial for maintaining cell-autonomous circadian rhythm in the SCN.

### Molecular Mechanisms of the Circadian Clock

In mammals, the circadian clock is mainly composed of three transcriptional-(post)translational feedback loops (Figure 1). Transcription factors BMAL1 and CLOCK heterodimerize and activate transcription of the clock genes *Period (PER1, PER2)* and *Cryptochrome (CRY1, CRY2)*. As they increasingly accumulate in the cytosol, PER and CRY heterodimerize, translocate into the nucleus, and shut down the expression of their own genes by inhibiting BMAL1-CLOCK. The turnover and activity of these core clock proteins are controlled by post-translational modifications, including phosphorylation, ubiquitination, acetylation, and O-linked β-*N*-acetylglucosaminylation (*O*-GlcNAcylation) (Panda, 2016; Takahashi, 2017). Phosphorylation of PER2 serine 662 is a key regulatory node in setting the speed of the clock, initiating the casein kinase 1δ/ε-controlled phosphorylation events and ubiquitin-mediated protein degradation.

BMAL1-CLOCK also activates the expression of *REV-ERB*α *(NR1D1)* and *REV-ERB*β *(NR1D2)*, which repress expression of *BMAL1* and *NFIL3* (nuclear factor, interleukin 3-regulated, also known as E4BP4). Retinoic acid-related orphan receptor (ROR) and REV-ERB competitively bind to RORE *cis*-elements, resulting in the regulation of one stabilizing loop of the circadian clock. The other stabilizing loop is composed of the PAR-bZIP (proline and acidic amino acid-rich basic leucine zipper) factors DBP (D-box binding protein), TEF (thyrotroph embryonic factor), and HLF (hepatic leukemia factor), whose expression is controlled by BMAL1-CLOCK. REV-ERB/ROR-controlled repressor NFIL3 and the PAR-bZIP transcription factors regulate expression of genes containing D-box *cis*-elements. Together, these three interlocking feedback loops generate robust circadian rhythms of the clock genes and output genes, whose phase and amplitude are determined by combinations of circadian *cis*-regulatory elements in the promoter and enhancer regions.

## Circadian Physiology in Major Organs Associated With Complex Disease

### Liver

The liver is a prototypical peripheral organ that is deeply regulated by the circadian clock system. Both transcriptional and organelle-level mechanisms orchestrate diurnal rhythms of liver physiology, ranging from the well-characterized metabolic functions (including metabolism of glucose, glycogen, lipids, and amino acids) to cell growth and xenobiotic responses. For example, the circadian clock drives diurnal expression of glycogen synthase 2 (GYS2) to modulate glycogen synthesis, phosphoenolpyruvate carboxykinase 1 (PEPCK1) and glucose-6-phosphatase, catalytic (G6PC) for gluconeogenesis; and insulin induced gene 2 (INSIG2) for cholesterol and bile acid synthesis (Bass and Lazar, 2016; Panda, 2016; Reinke and Asher, 2019; Zhang et al., 2020b). PER2 and CRY1 bind to nuclear hormone receptors, such as REV-ERBα/β, PPARα, and GR to output the diurnal rhythms to glucose and lipid metabolism (Lamia et al., 2008; Schmutz et al., 2010; Zhang et al., 2010). Emerging evidence suggests a SCN-liver axis in the control of diurnal rhythm in hepatic glucose metabolism. REV-ERBα/β in SCN GABAergic neurons are required for rhythmic insulin-mediated suppression of glucose production in the liver (Ding et al., 2021). In addition to cell-autonomous effects, the hepatocyte clock can modulate the circadian rhythm of local microenvironment. Hepatic REV-ERBα/β is required for the circadian rhythmicity of transcriptomes and metabolomes in local endothelial cells and Kupffer cells in the liver, which is associated with circadian rhythms of glutathione metabolism, glycolysis, and hexosamine pathway (Guan et al., 2020).

It has become apparent that clock proteins can execute temporal order through crosstalk to organelle structure and dynamics. To meet the daily demand of cellular energy and stress adaptation, mitochondrial structure and organization are regulated by the circadian clock. Hepatic BMAL1 is crucial for circadian rhythm of the mitochondrial fission, fusion, and turnover by autophagy, in part through its transcriptional target genes involved in mitochondrial fission and fusion (Jacobi et al., 2015). Nutrient-sensing pathways such as AMPK and Sirtuins drive circadian rhythm of phosphorylation of mitochondrial fission executor, dynamin-related protein 1 (DRP1, peak at CT 12 h). This in turn leads to diurnal oscillations of mitochondrial morphology between fission and fusion and cellular energy (Schmitt et al., 2018). In synchrony with the cycling of mitochondrial structure, mitochondrial fatty acid beta-oxidation (peak at ZT0) and protein abundance of mitochondrial rate-limiting enzymes (CPT1A peak at ZT 0 h and PDHB peak at ZT 3 h) exhibit diurnal rhythms, which are all controlled by PER1/PER2 (Neufeld-Cohen et al., 2016).

Lysosomes exhibit diurnal activities in the liver. Autophagy promotes lysosomal degradation of CRY1, contributing to clock regulation (Toledo et al., 2018). Using p62/SQSTM1 as a marker, Ryzhikov et al. (2019) found that basal autophagosome formation (peak at ZT 8 h) is coupled to proteasomal protein degradation (peak at ZT 10 h) during the resting phase. Interestingly, proinflammatory signals such as lipopolysaccharide (LPS) alter the substrate preference of autophagy from cytosolic proteins to mitochondrial proteins (Ryzhikov et al., 2019). Diurnal expression of autophagic genes is conferred in part through diurnal transcriptional activity of CCAAT enhancer binding protein beta (C/EBPβ) (Ma et al., 2011). Emerging evidence supports the strong circadian regulation of autophagy at the proteomic and post-translational levels. Wang Y. et al. (2018) showed that lysosomal transport signature is enriched in diurnal proteome in liver, however, there is no circadian rhythm of the transcripts encoding these proteins. Autophagic proteins and their regulator, mammalian target of rapamycin complex 1 (mTORC1), which is among the 25% of the hepatic circadian phospho-proteome, exhibit circadian rhythm in liver (Robles et al., 2017). Cytosolic PER2 tethers tuberous sclerosis 1 protein (TSC1) to suppress the activity of mTORC1, contributing to diurnal rhythms of protein synthesis and autophagy in liver (Wu et al., 2019). To date, the majority of evidence suggesting clock-output pathways to lysosomes and autophagosomes is based on biochemical markers. Compelling evidence from microscopy of subcellular structure would help establish the links between lysosome/autophagy and circadian clocks in the liver and other tissues.

### Heart and Skeletal Muscle

#### Heart and the Cardiovascular System

The heart and cardiovascular system exhibit circadian rhythm of genes and functions from the heart tissue to various types of blood vessels (Crnko et al., 2019). Heart tissue is a specialized mechanic pump in the body, which creates a unique organ system where two physiological cycles interact like a Russian doll. Namely, the second-scale cardiac cycle is gauged by the circadian clock. It is estimated that 13% of gene transcripts and 8% of proteins in the mouse heart are diurnal (Martino et al., 2004; Podobed et al., 2014). Under constant darkness, a lower percentage (6–8%) of gene transcripts oscillate in a circadian manner (Storch et al., 2002; Zhang et al., 2014). In the aorta, 4–5% of genes oscillate in a circadian manner (Rudic et al., 2005; Zhang et al., 2014). These circadian pathways span from cellular energy metabolism and sarcolemma calcium signaling, to cellular signaling pathways.

The cardiomyocyte clock is crucial in driving the cell-autonomous oscillation of heart function (Durgan et al., 2005; Bray et al., 2008). Mitochondrial function and dynamics sit at the hub of this regulation (Zhang et al., 2020a). Intervening in CLOCK function by overexpressing a dominant-negative mutant abolishes diurnal variation of mitochondrial oxidative metabolism (peak ZT 18h) and tolerance to ischemic-reperfusion injury (peak ZT 0 h) (Durgan et al., 2005, 2010; Bray et al., 2008). These phenotypes are associated with the abolished oscillation of PDK4, a metabolic gauge between glycolysis and oxidative metabolism. Genetic deletion of the *Bmal1* gene in mouse cardiomyocytes results in dilated cardiomyopathy, decreased heart rate and increased arrhythmias (Lefta et al., 2012; Schroder et al., 2013). In human embryonic stem cell-derived cardiomyocytes, loss of BMAL1 inhibits mitochondrial fission and mitophagy, impairs oxidative metabolism, and leads to disorganized sarcolemmal structure, decreased contractility, and dilated cardiomyopathy (Li E. et al., 2020). Mechanistically, BMAL1/CLOCK bipartite TF trans-activates expression of the mitophagy receptor gene BNIP3 (Li E. et al., 2020). Provided that BNIP3 is not a robust diurnal gene in the mouse heart (source: CirGRDB, CircaMetDB), it remains open to post-translational or organelle-level regulation of mitochondria which contributes to diurnal oscillation of the cardiac cycle and metabolism in the heart.

**FIGURE 2:**
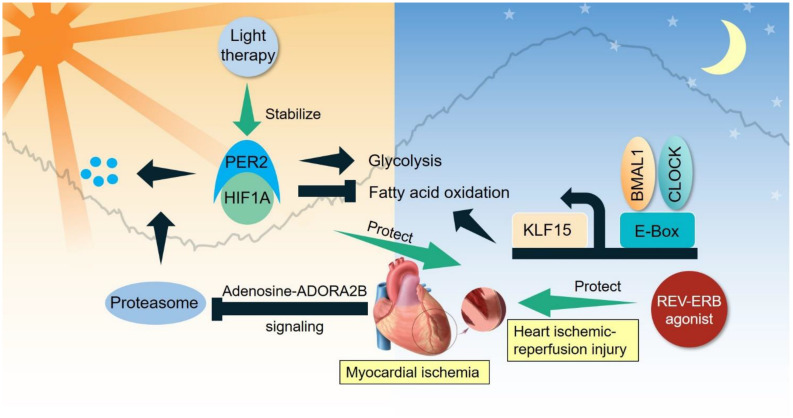
Clock-controlled checkpoints in the heart. Myocardial ischemia stabilizes PER2 to promote glycolysis and suppress fatty acid oxidation. BMAL1/CLOCK bipartite TF modulate the diurnal oscillation of fatty acid oxidation partly through KLF15. Light therapy stabilizes PER2 to benefit ischemic-reperfusion injury, while REV-ERB agonist also protects against ischemic-reperfusion injury.

Moreover, clock-controlled transcription factors orchestrate the cardiac cycle and metabolism (Figure 2). Krüppel-like factor 15 (KLF15), a BMAL1-controlled output TF, determines diurnal oscillation of the cardiac cycle (peak ZT 6 h) and susceptibility to ischemic-reperfusion injury to the heart (Jeyaraj et al., 2012; Li L. et al., 2020). The cardiac arrhythmogenesis in KLF15 transgenic animals phenocopies that in an inducible cardiomyocyte-specific *Bmal1* knockout model (Schroder et al., 2013). Though initially thought to act through diurnal expression of ion channels, transcriptome profiling demonstrates that KLF15 controls the diurnal oscillation of cellular energy metabolism (peak ZT 14–20 h) and tissue remodeling and repair (peak ZT 0–6 h) (Zhang et al., 2015). Compelling evidence links the activity of oxygen-sensing HIF-1α transcription factor to the heart-clock, which outputs the diurnal control of glycolysis and the hypoxia response (Eckle et al., 2012; Wu et al., 2017). Chemical activation of REV-ERBα protects against heart failure, which is partially linked to transcription repression (Zhang et al., 2017). While it is intriguing to propose TF-specific labor division in organizing various aspects of the cardiac cycle, contractility, and metabolism throughout the day, it remains unsolved how these clock-output TFs forge a network to coordinate diurnal oscillation of heart physiology and metabolism.

#### Skeletal Muscle

**FIGURE 3:**
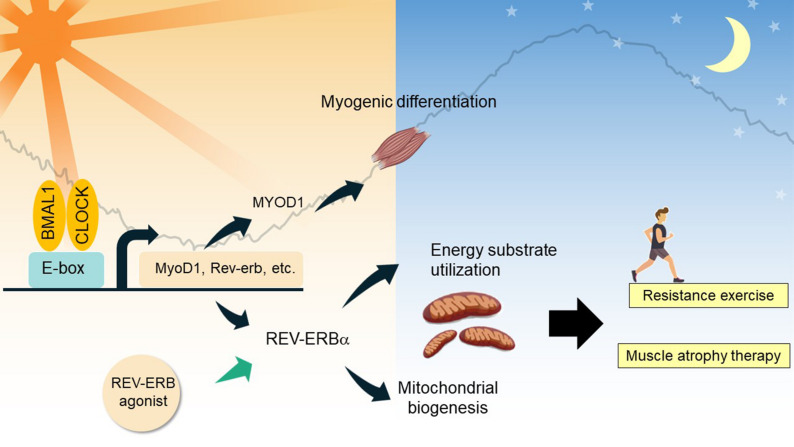
Clock-controlled checkpoints in skeletal muscle. In myocytes, BMAL1 and CLOCK regulate diurnal expression of genes involved in myogenic differentiation and energy substrate utilization. REV-ERBα and its interactome regulate expression of mitochondrial biogenic genes and energy substrate utilization, contributing to myofiber type switch. As demonstrated in chemical biology studies, REV-ERBα agonism holds promise to treat skeletal muscle atrophy, and improve resistance exercise performance.

Skeletal muscle is the most abundant tissue in mammals, comprising approximately 40% of body mass. Similar to other tissues, skeletal muscle also possesses its own self-sustaining endogenous molecular clock. Approximately 20% of genes (>2300 genes) show 24 h oscillations in skeletal muscle, and these genes participate in a wide range of functions, including myogenesis, transcription and metabolism (Chatterjee and Ma, 2016). Recently, the importance of circadian rhythms for skeletal muscle has been made more evident by the skeletal muscle phenotype observed in models of molecular clock disruption (Figure 3).

Firstly, the circadian clock network plays a prominent role in muscle differentiation and maintenance. A study of *Bmal1*-null mice clearly shows atrophy and tissue damage in skeletal muscle, which could be attributed to the blunted circadian expression of MyoD1 (myogenic differentiation 1) and a defect in the regenerative myogenic process accompanied by lowered repair and an altered satellite cell expansion process (Schiaffino et al., 2016; Vitale et al., 2019). Clock nuclear receptors REV-ERBα/β are highly expressed in proliferating myoblast cells and repress key genes involved in myotube differentiation (Everett and Lazar, 2014; Chatterjee and Ma, 2016). Schroder et al. (2015) investigated a *Bmal1* conditional knockout line in adult skeletal muscle, and showed that remarkably, this model mimics the advanced aging phenotype in the musculoskeletal system in Bmal1-null mice, characterized by increased fibrosis, bone calcification, and decreased joint collagen. Skeletal muscle BMAL1 exclusively controls diurnal expression of core clock genes and nuclear receptor genes (Dyar et al., 2014; Schroder et al., 2015). CRY2 regulates the circadian rhythm of myogenic differentiation through stabilization of Cyclin D1 and Tmem176b transcripts (Lowe et al., 2018).

Secondly, the circadian clock participates in the temporal control of energy metabolism in skeletal muscle and contributes to systemic metabolism. Early studies indicate that metabolic genes involved in substrate utilization and energy storage are expressed in a temporally segregated manner over a 24-h period, suggesting a clock-controlled orchestration of distinct catabolic and anabolic metabolic pathways in skeletal muscle (Chatterjee and Ma, 2016; Gabriel and Zierath, 2019). Further, a number of studies have demonstrated the mechanisms underlying the interplay between the molecular clock and fuel preference in cellular energy metabolism (Dyar et al., 2014; Mayeuf-Louchart et al., 2015; Perrin et al., 2018). Muscle-specific *Bmal1* knockout resulted in a clear up-regulation of genes involved in lipid metabolism concomitant with the down-regulation of circadian genes involved in glucose utilization, indicating muscle fiber type switches to a slow oxidative fiber-type, together with a substrate shift from carbohydrate to lipid utilization (Schiaffino et al., 2016). BMAL1 also controls HIF-1α activity in myocytes, contributing to the regulation of anaerobic glycolysis (Peek et al., 2017). REV-ERBα is highly expressed in oxidative fiber types, which promotes oxidative metabolism and ultimately exercise performance, in part through the nutrient-sensing LKB-AMPK-SIRT1-PGC1α signaling complex (Woldt et al., 2013). Increased fatty acid utilization, hyperglycemia, and a clear fast-to-slow MyHC in soleus muscle were observed in *Rev-Erbα*-deficient mice (Woldt et al., 2013), whereas activation of REV-ERBα by synthetic agonists resulted in lipid lowering and anti-obesity effects (Cho et al., 2012).

Thirdly, the circadian clock has strong control of muscle strength and locomotor function. Results from circadian transcriptomics studies indicate that many essential functions and physiological processes in skeletal muscle are influenced by the transcriptional output of the clock (Dyar et al., 2018a; Perrin et al., 2018). As observed in mice, a high proportion of cycling transcripts peak midway through the dark phase, coinciding with the peak period of physical activity and feeding in nocturnal species. Clock genes in the negative limb such as CRY and PER, control the diurnal rhythm of exercise performance through regulation of energy substrate utilization (Jordan et al., 2017; Ezagouri et al., 2019). Histone deacetylase 3 (HDAC3), the interacting partner of clock proteins including REV-ERB, also governs exercise performance (Hong et al., 2017). Meanwhile, a chronotype effect on the circadian expression of many types of physical performance has also been observed (Chatterjee and Ma, 2016; Vitale et al., 2019). For example, optimal human muscle torque, strength and power are generally displayed in the late afternoon but not in the morning, suggesting that locomotor activity may coordinate the phase of the intrinsic rhythmic expression of genes in skeletal muscle.

Besides the above mentioned circadian regulation on skeletal muscle, physical activity could function as a strong clock entrainment signal, particularly for the skeletal muscle clock (Sato et al., 2019). Resistance exercise is capable of shifting the expression of diurnally regulated genes in human skeletal muscle (Zambon et al., 2003). Loss of muscle activity leads to marked muscle atrophy and reduced expression of core clock genes in mouse skeletal muscle (Zambon et al., 2003). Overall, recent findings demonstrate the intimate interplay between the cell-autonomous circadian clock and muscle physiology.

### Blood

Many parameters in blood exhibit circadian rhythmicity, including leukocytes, erythrocytes, chemokines (e.g., CCL2, CCL5), cytokines (e.g., TNFα, IL-6), and hormones (Schilperoort et al., 2020). The most apparent oscillation in blood is observed in the number and type of circulating leukocytes, which peak in the resting phase and reach a trough in the activity phase during 24 h in humans and rodents (He et al., 2018). This time-dependent alteration of leukocytes reflects a rhythmic mobilization from hematopoietic organs and the recruitment process to tissue/organs (Méndez-Ferrer et al., 2008; Scheiermann et al., 2012). For example, the mobilization of leukocytes from the bone marrow is regulated by photic cues which are transmitted to the SCN and modulate the microenvironment of the bone marrow through adrenergic signals (Méndez-Ferrer et al., 2008).

Leukocytes exit the blood by a series of interactions with the endothelium, which involves various adhesion molecules, chemokines and chemokine receptors (Vestweber, 2015). Using a screening approach, He et al. (2018) depicted the time-dependent expression profile of the pro-migratory molecules on different endothelial cells and leukocyte subsets. Specific inhibition of the promigratory molecule or depletion of Bmal1 in leukocyte subsets or endothelial cells can diminish the rhythmic recruitment of the leukocyte subset to tissues/organs, indicating that the spatiotemporal emigration of leukocytes is highly dependent on the tissue context and cell-autonomous rhythms (Scheiermann et al., 2012; He et al., 2018). Cell-autonomous clocks also control diurnal migration of neutrophils (Adrover et al., 2019), Ly6C-high inflammatory monocytes (Nguyen et al., 2013) in the blood and leukocyte trafficking in the lymph nodes (Druzd et al., 2017).

**FIGURE 4:**
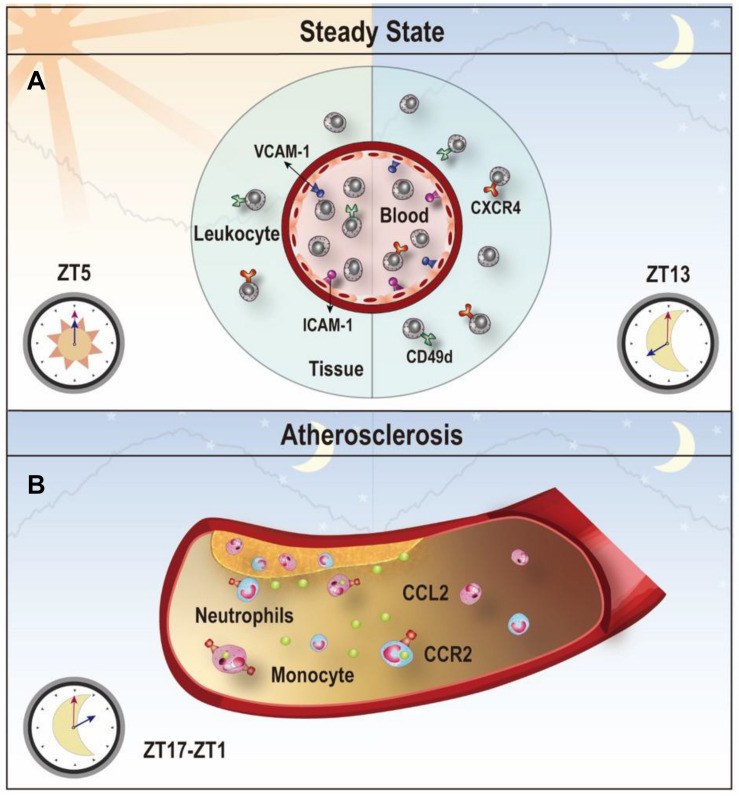
Clock-controlled checkpoints in leukocyte migration. **(A)** Leukocyte migration is controlled by the circadian clock. Rhythmic expression of promigratory molecules, such as ICAM-1, VCAM-1, CD49d, and CXCR4, promotes migration and retention of leukocytes to tissues, which peaks at ZT5 in the steady state. **(B)** Chemokine CCL2-CCR2 signaling is a clock-controlled checkpoint in leukocyte migration under atherosclerosis. Myeloid cells adhere to atherosclerotic lesions in a rhythmic manner with a peak between ZT17-ZT1 because of the diurnal expression of the CCL2-CCR2 axis. Targeting the CCL2-CCR2 axis in this time period may reduce inflammation during atherogenesis.

Furthermore, the circadian recruitment process of leukocytes was not only found in the steady state but also in some pathologic states, such as natural aging (Adrover et al., 2019), the LPS-induced inflammatory scenario (He et al., 2018), and parasite infections (Hopwood et al., 2018). These findings suggest that leukocyte migration retains a circadian rhythmicity in response to pathogenic insults. Although mammalian erythrocytes lack the genetic oscillator, the peroxiredoxin system in erythrocytes has been shown to follow 24-h redox cycles (O’Neill and Reddy, 2011). Moreover, the membrane conductance and cytoplasmic conductivity of erythrocytes exhibit circadian rhythmicity depending on cellular K++ levels (Henslee et al., 2017). These observations indicate that non-transcriptional oscillators can regulate the circadian rhythms in denucleated cells. In addition to leukocytes and erythrocytes, other parameters in blood like chemokines and cytokines also exhibit a circadian rhythmicity (Schilperoort et al., 2020). Together, emerging evidence shows that the circadian rhythm can be easily found in blood elements which are essential contributors to the maintenance of circadian physiology (Figure 4A).

### Peripheral and Central Nervous System

**FIGURE 5:**
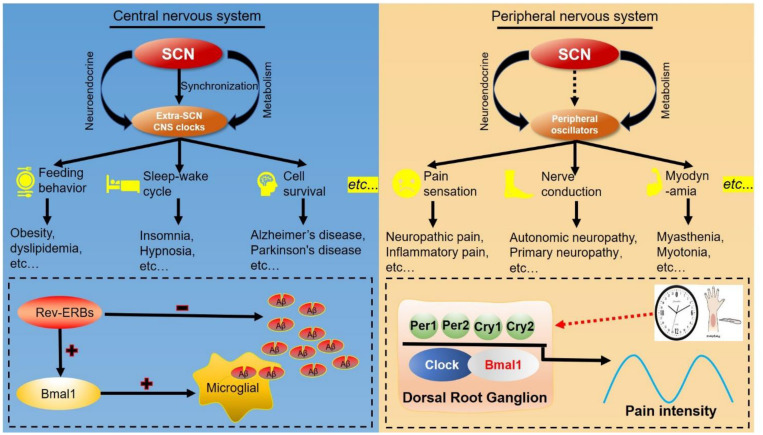
Clock-controlled checkpoints in neurons. The suprachiasmatic nucleus (SCN) synchronizes extra-SCN clocks and peripheral oscillators in the central nervous system (CNS) and peripheral nervous system. **Left:** The SCN master oscillator and its output extra-SCN CNS clocks control diverse functions in the central nervous system, including feeding behavior, sleep–wake cycles and cell survival. For example, REV-ERB decreases the number of amyloid-beta (Aβ) and increases BMAL1 transcription to accelerate microglial uptake of Aβ. **Right:** Peripheral oscillators can regulate functions and diseases of the peripheral nervous system, such as pathological pain. Rhythmic expression of BMAL1 and PER2 in dorsal root ganglions modulate the response to noxious stimulation, and establish the circadian rhythm of pathological pain.

Circadian clock directs multiple metabolic and physiological functions in both the peripheral and central nervous system (Figure 5). In the central nervous system, many physiological processes controlled by extra-SCN hypothalamic nuclei display diurnal rhythms, such as those involved in energy and temperature regulation, glucose and lipid metabolism (Paul et al., 2020). Clocks in the forebrain, arcuate nucleus and dorsomedial hypothalamus can integrate external cues including temperature and nutrition cycles. Complete loss of circadian behavior was found in forebrain/SCN-specific Bmal1 knockout mice, and the related circadian rhythms in peripheral tissues was differentially affected by light/dark cycles and feeding (Izumo et al., 2014). Time-restricted feeding in mice has been shown to impair the body temperature homeostasis (Zhang et al., 2020c). Circadian gene expression analysis in the dorsomedial hypothalamus revealed that rhythmically reprogramming of thermoregulation gene expression is involved in the impairment of body temperature regulation (Zhang et al., 2020c). Integrative cistromic and transcriptomic analysis showed that REV-ERB-dependent leptin signaling in the arcuate nucleus plays an important role in the control of diurnal leptin sensitivity and food intake in diet-induced obesity (Adlanmerini et al., 2021). With more and more neuronal circadian oscillators uncovered, circadian rhythms of the circuit-level communication, organization, and physiological functions need to be explored.

The hypothalamus-pituitary-adrenal (HPA) axis is a major neuroendocrine pathway involved in stress response, metabolism, and circadian rhythm. HPA is regulated in a circadian manner, and peaks in the dawn in humans, or in the onset of the dark phase for nocturnal animals (Oster et al., 2017). Rhythmic release of the inhibitory neuropeptide, vasopressin, from the SCN periodically inhibits corticotrophin-releasing hormone (CRH)-neurons in the paraventricular nucleus (PVN) of hypothalamus. Vasopressin may reach the CRH neurons of PVN via either extracellular space/fluid or direct neuronal projection (Vrang et al., 1995; Tousson and Meissl, 2004). Release of CRH into the anterior pituitary promotes the release of adrenocorticotrophic hormone (ACTH) into the blood. Plasma ACTH is circadian, however, ACTH precursor Pomc does not oscillate at the transcript level in the anterior pituitary (Oster et al., 2017). ACTH reaches the adrenal gland and promotes secretion of glucocorticoids to the blood. The adrenal-clock controls circadian and acute secretion of glucocorticoids (Leliavski et al., 2014). *Bmal1*-deficiency decreases the diurnal rise of plasma glucocorticoids around the onset of the dark phase. *Bmal1*-decificency abolishes stress-induced rise of plasma glucocorticoids, but does not affect stress-induced rise of plasma ACTH. PER2 governs the diurnal rise in plasma glucocorticoid at the onset of the dark phase (Yang et al., 2009; Russell et al., 2021). PER2 does not regulate glucocorticoid secretion towards major stressors, such as hypoglycemia, ACTH, and physical restraint, neither does PER2 regulate ACTH peptide rhythm in the hypothalamus (Yang et al., 2009). Together, these findings demonstrate that both the SCN-clock and the adrenal-clock control the circadian activity of the HPA axis.

The peripheral nervous system mainly consists of sensory neurons, and their primary function is to transmit the sensory signals such as pain, touch, itch, and temperature sensation (Niesler et al., 2021). Pain sensitivity follows a daily cycle in many clinical conditions, and there is strong evidence to support the rhythmicity in response to nociceptive stimuli (Bruguerolle and Labrecque, 2007). The processing of painful stimulation occurs in the dorsal horn (DH), an area of the spinal cord that receives noxious stimulation signals from peripheral tissues via several types of primary afferent nerve fibers (Crodelle et al., 2019). Pain perception exhibits a 24-h rhythm regardless of whether pain threshold is objectively or subjectively assessed (Burish et al., 2019). Mechanistically, BMAL1, CLOCK, PER1, and REV-ERBα contribute to neuropathic pain at evening and cluster headache at midnight (Burish et al., 2019). Core clock genes are rhythmically expressed in neurons of the dorsal root ganglion (Kim H. K. et al., 2020). A Per2 mutation abolishes the circadian rhythm of the inflammatory pain response (peak ZT4) (Zhang et al., 2012b). Together, these studies provide evidence to support a potential strategy for improvement of pain treatment based on the circadian rhythm. More detailed studies are required to explore this phenomenon and more clinical trials needed to validate these findings.

### Male Reproductive System

Diurnal rhythms of sperm count, sperm motility, and sperm chromatin integrity have been found in rodents. There are also several studies which have investigated the diurnal variation of semen parameters in humans (Ni et al., 2019; Sati, 2020). Variations in semen parameters were found across different time points in most of the studies, although a high-resolution sampling study is required to confidently profile the circadian pattern. It is also well known that semen parameters have circannual variation both in men and male animals (Chemineau et al., 2008; Xie et al., 2018). These studies suggest that the circadian clock and its regulatory mechanisms may play an important role in the regulation of the male reproductive system.

**FIGURE 6:**
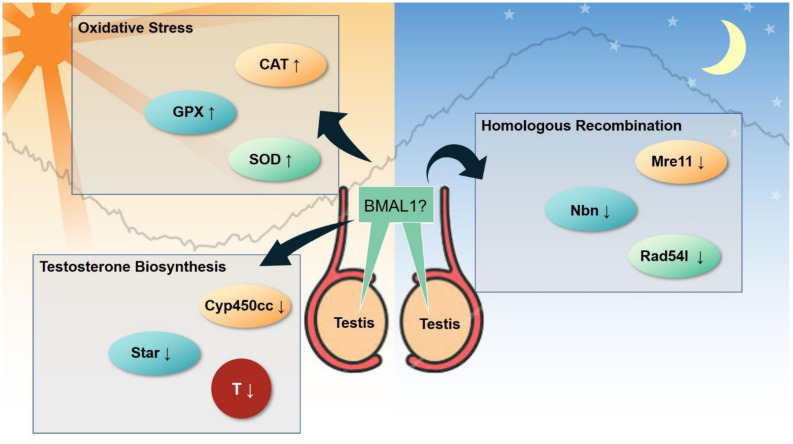
Emerging clock-controlled checkpoints in the testis. Emerging evidence suggests the role of the core clock protein BMAL1 in the regulation of oxidative stress, homologous recombination and testosterone biosynthesis in testis, which may potentially contribute to the pathogenesis of circadian-related disorders. CAT, catalase. GPx, glutathione peroxidase. SOD, superoxide dismutase. Cyp450cc, cytochrome P450 side-chain cleavage. Star, steroidogenic acute regulatory protein. T, testosterone. Mre11, double strand break repair nuclease. Nbn, nibrin. Rad54l, RAD54 like.

Clock genes are expressed in different parts of the male reproductive system, including extra-testicular ducts and accessary organs. However, the presence of a cell-autonomous clock in testes remains controversial (Figure 6; Alvarez et al., 2003; Mazzoccoli et al., 2012). In insects, *per* mRNA in testes oscillate under light-dark conditions, but the diurnal rhythm is not self-sustaining under constant darkness (Gvakharia et al., 2000). Leydig cells, the primary androgen-secreting cells in testes, express BMAL1 in a circadian manner, however, the mRNA levels of clock genes are not diurnal in testes (Chen et al., 2017). Dexamethasone synchronizes the expression of several circadian clock genes and steroidogenic-related genes in Leydig cells *in vitro* (Chen et al., 2017). Expression of PER1 protein is strictly isolated to certain stages of spermatogenesis, i.e., spermatogonia and condensing spermatids, while the expression of CLOCK is restricted to round spermatids (Alvarez et al., 2003). In the diurnal transcriptome atlas for major neural and peripheral tissues of the Papio anubis (baboon), 1672 cycling genes were identified in testes (Mure et al., 2018). However, the core clock genes such as *CLOCK, BMAL1, PER1-3, CRY1-2*, and *RORA* were not in the list. It is likely that a cell-autonomous clock is restricted to specific cell types such as the Leydig cell. The molecular analysis using bulk tissues masks the rhythmicity since clock genes may be expressed ubiquitously in different types of cells including the clock-less cells. The use of single-cell omics in circadian studies would resolve this issue since these techniques have already been applied in the testes (Guo et al., 2018; Lau et al., 2020; Shami et al., 2020).

Clock genes are essential for the development of the male reproductive system. Knockout of the Bmal1 gene induces infertility and decreases the production of testosterone in mice (Alvarez et al., 2008). Bmal1-deficient male mice seem to have defects in copulatory behaviors (Schoeller et al., 2016). Interestingly, if Bmal1 was conditionally knocked out during adult life, the fertility of the mice was conserved (Yang et al., 2016). Circadian clock becomes functional at around embryonic days 13–18 (Umemura and Yagita, 2020). It has to be determined whether Bmal1 has a non-clock function on the male reproduction or that the testes-clock is required for the male reproduction before sex maturity. If induced deletion of Bmal1 on embryonic days 13–18 still preserved fertility in males, it would clearly support the non-clock function of BMAL1 in male fertility. Knockdown of Clock in mouse testes leads to reproductive damages, such as small litter size, decreased *in vitro* fertility rate, blastula formation rate, and acrosin activity of the sperm (Liang et al., 2013). Mechanistically, BMAL1 and CLOCK regulate the assembly of chromatoid bodies through interaction with MVH, a key component of the sperm chromatoid body (Peruquetti et al., 2012). A deficiency of Cry1 was found to increase apoptosis of mouse testicular germ cells and decrease sperm count (Li C. et al., 2018). Sertoli cells provide a nurturing and supportive niche for spermatogenesis. The specific knockdown of Rora in Sertoli cells at puberty leads to declined sperm count in rats (Mandal et al., 2018). In *C. elegans*, NHR-23/NR1F1 (RORA) depletion causes infertility due to the arrest of primary spermatocytes rather than haploid sperm (Ragle et al., 2020).

Disruption of circadian cycles is associated with high activity of the hypothalamic-pituitary-adrenal (HPA) axis. Lassi et al. (2021) showed that inverted feeding (night restricted feeding, of which night refers to the light phase) leads to dampened rhythm and high circulating levels of corticosterone in male founder mice. By carefully controlling the mating and breeding procedure, Lassi et al. (2021) demonstrated that paternal circadian disruption by inverted feeding can be transmitted to male offspring through hyper-corticosteronemia at conception. Hyper-corticosteronemia at conception resulted in a plethora of metabolic abnormalities in the male offspring, such as hyperphagia, hyper-metabolism, hyperglycemia, and hyper-corticosteronemia (Lassi et al., 2021). This trans-generational transmission of paternal circadian disruption does not seem to be pathogenic, as body weight, glucose tolerance, and insulin levels remained normal. Interestingly, wildtype male offspring of Clock mutant males recapitulate the high energy metabolism, indicating that inverted feeding imprints the high HPA activity in male offspring in part through the circadian clock (Lassi et al., 2021). Thus, paternal glucocorticoid signaling at conception may transmit the effects of circadian disruption to the offspring.

In humans, genetic polymorphisms of circadian genes have been linked to fertility and semen quality. Three single nucleotide polymorphisms (SNPs) of the Clock gene (rs11932595, rs6811520, and rs6850524) were associated with the risk of infertility in a case-control study of 961 Slovenian and Serbian Caucasian men (Hodžić et al., 2013). Another two Clock SNPs (rs1801260 and rs3817444) were found to correlate with infertility risk in 672 Chinese men (Shen et al., 2015). Several SNPs were also associated with semen volume, sperm count, sperm motility, as well as the levels of testosterone and follicle-stimulating hormone (Zhang et al., 2012a; Shen et al., 2015). In an observational study of 106 university students, levels of testosterone correlated with scores on the Composite Scale of Morningness, an indicator of chronotype, but not with sleep duration (Randler et al., 2012). However, it is not clear whether there is substantial damage to male fertility in syndromes related to clock gene mutations, such as Familial Advanced Sleep Phase Syndrome.

### Female Reproductive System: Placenta

**FIGURE 7:**
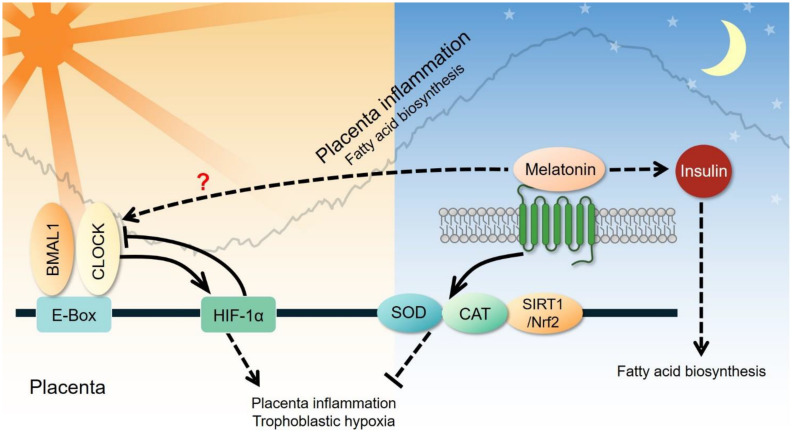
Clock-controlled checkpoints in the placenta. Placental inflammation and trophoblastic hypoxia are promoted by the CLOCK-HIF-1α signaling pathway during the active phase and suppressed through the melatonin-superoxide dismutase (SOD)-catalase (CAT) signaling pathway during the rest phase. However, the exact relationship between circadian genes and melatonin remains unclear. Melatonin can also elevate plasma insulin and subsequently increase fatty acid biosynthesis in the placenta.

The placenta is an organ that functions to exchange nutrients, gasses, wastes, and hormones between the mother and fetus. Growing evidence suggests the presence of a circadian clock in the placenta (Figure 7). Hypoxia has been shown to induce circadian expression of *PER2* and *DEC1* genes in human placental cells (Frigato et al., 2009). Transcript levels of clock genes *BMAL1*, *CLOCK*, *CRY1-2*, and *PER1-2* oscillate in the murine gravid uterus and placenta during late gestation (Ratajczak et al., 2010). Placental function also expresses a daily rhythm, as observed in the maternal plasma human chorionic gonadotropin (hCG) levels during early pregnancy, at the time of maximum placental hCG expression (Díaz-Cueto et al., 1994). Pérez et al. (2015) revealed the first piece of evidence demonstrating circadian expression of *CLOCK*, *BMAL1*, *PER2*, and *CRY1* genes in the human full-term placenta. Clarkson-Townsend et al. (2020) found seasonal rhythmicity of *DEC1* in human full-term placentas, adding evidence for the placenta as a peripheral clock. While clock mutant mice exhibit defects in female fertility and growth retardation associated with the placenta, the standard transcriptional/translation feedback loops of the oscillator mechanism in placenta appear less coordinated and robust. Rather, one study showed *BMAL1* and *PER* rhythms are of low amplitude and not anti-phasic, suggesting a weak, if any, functioning of the core clock in the placenta (Wharfe et al., 2011). As of now, it is premature to conclude that a self-sustaining circadian clock is present in the placenta. It is likely that only certain regions of the placenta have a functional clock. Demarez et al. (2021) showed that the trophoblast layer of the labyrinth zone has a functional clock by late gestation, which controls diurnal expression and activity of ABCB1, a xenobiotic efflux pump. A simple bioinformatics survey in the Human Protein Atlas database revealed strikingly high levels of core clock proteins in the placenta, such as *PER2*, *CRY1*, *BMAL1.* Together, these studies provide justification for the need to explore cell type-specific clocks in the placenta and link the circadian clock to the nexus of maternal-fetal communication.

Furthermore, melatonin and steroid hormones are known to follow circadian pattern due to the diurnal activity of the pineal gland and the hypothalamus-pituitary axis (Urlep and Rozman, 2013). Melatonin, a pineal gland secreted factor, is secreted mainly during the nighttime of the diurnal cycle. Melatonin plays an essential role in synchronizing the peripheral tissues to the 24-h circadian rhythms and likely provides feedback to the hypothalamus-pituitary-adrenal axis and the hypothalamus-pituitary-gonad axis (Shi L. et al., 2013; Huang et al., 2020). Melatonin can regulate the expression of clock genes in the placenta via its melatonin receptors (Lanoix et al., 2006), and this may contribute to adverse pregnancy outcomes (Lanoix et al., 2012; Olcese et al., 2013; Shimada et al., 2016).

## Circadian Pathophysiology in Complex Diseases

### Metabolic Associated Fatty Liver Disease

Metabolic associated fatty liver disease (MAFLD), formerly known as non-alcoholic fatty liver disease, is the liver manifestation of metabolic syndrome, and affects 25% of the global population (Bayoumi et al., 2020). MAFLD is the beginning stage of a continuum of chronic liver diseases, which ends with cirrhosis, liver failure, or hepatocellular carcinoma (Friedman et al., 2018). MAFLD is characterized by fat-loaded hepatocytes without signs of liver injury. Recent epidemiological studies have revealed a clear association between circadian rhythm disorder and the incidence of MAFLD (Peng et al., 2017; Wang H. et al., 2018; Hu et al., 2020). Clock mutant mice are prone to liver steatosis when fed a high-fat diet, which was able to be restored to homeostasis by time-restricted feeding (Chaix et al., 2019; Saran et al., 2020; Zhang et al., 2020b). The vast majority of hepatic triglycerides in the human liver come from adipose-released fatty acids (59%), followed by de novo lipogenesis (26%), and the diet (15%) (Francque et al., 2020). Studies of germline, liver-specific, and adipose-specific deletion of core clock genes indicate that core clocks contribute to liver steatosis mainly through extra-hepatic organs, such as the hypothalamus and adipose (Turek et al., 2005; Paschos et al., 2012; Zhang et al., 2020b), and may be dependent on exposure to circadian insult in early life (Yang et al., 2016).

Nuclear receptors and other non-clock transcription factors are emerging as regulators of diurnal lipid metabolism involved in the pathogenesis of MAFLD. Twenty of the 49 nuclear receptor genes exhibit a diurnal rhythm in the liver, providing the first line of transcription factors linking metabolism and clock (Yang et al., 2006). It has been well-established that REV-ERB and the co-repressor HDAC3 orchestrate diurnal rhythms of lipogenesis, and bile acid production (Bass and Lazar, 2016; Panda, 2016). Peroxisome-proliferating activator receptors (PPARα/β/γ) promote remodeling of the diurnal lipid metabolism in liver. A high-fat diet induces de novo oscillation of thousands of transcripts through PPARγ (peak ZT 8 h) in the liver, and contributes to diurnal rhythms of genes involved in lipid storage and gluconeogenesis (Eckel-Mahan et al., 2013). PPARδ is required for the diurnal rhythm of hepatic lipogenesis, and release of a diurnal lipid phosphatidylcholine 18:0/18:1 (peak at ZT 18 h) that promotes muscle fatty acid uptake (Liu et al., 2013). A high-fat diet enhances the daily cycling of enhancer activities associated with two opposing transcription factors, i.e., PPARα for fatty acid oxidation and SREBP1c for de novo lipogenesis (Guan et al., 2018). PPARs promote resolution of inflammation in immune cells through tethering apart the pro-inflammatory signaling complex, such as nuclear factor-kappa B and AP-1 (Li and Yang, 2011). Myeloid cell-specific disruption of Per1/Per2 results in hepatic steatosis, inflammation, and liver injury (Xu et al., 2014). Introducing PPARγ2 transgene back to clock-less macrophages helps to resolve the exacerbation of inflammation (Xu et al., 2014). Currently, dual-, and pan-PPAR agonists are intensively investigated as potential therapeutics for chronic liver diseases (Francque et al., 2020).

### Cardiovascular Disease

#### Heart Disease

Heart diseases, including ischemic heart disease, diabetic cardiomyopathy, and heart failure, have a relationship to circadian clocks throughout their pathogenesis and prognosis (Crnko et al., 2019; Zhang et al., 2020a, b). Experimental models of heart disease, such as cardiac ischemia-reperfusion, pressure overload-induced cardiac hypertrophy, and diabetic cardiomyopathy, show clear disturbance of circadian clock oscillation in the heart (Young et al., 2001, 2002; Kung et al., 2007). Shift work increases the susceptibility to ischemic heart disease (Knutsson et al., 1986; Vetter et al., 2016), as does circadian disruption of behavioral rhythm in mice (Martino et al., 2007).

Ischemic heart disease is initiated by insufficient supply of blood (ischemia) to heart tissue due to obstruction of coronary arteries. Adaptive remodeling of heart metabolism is key to recovery and survival after ischemia (Sedej, 2018). Compelling evidence demonstrates several key clock-controlled checkpoints in heart metabolism that are crucial for treating ischemic heart disease. Myocardial ischemia induces adenosine-ADORA2B signaling that stabilizes PER2 through inhibition of proteasomal degradation (Eckle et al., 2012). PER2 promotes glycolysis and suppresses fatty acid oxidation in a HIF-1α-dependent manner, leading to reduced myocardial infarction. Interestingly, strong light exposure (10,000 lux) in the subjective day time stabilizes PER2 and protects the heart from ischemic-reperfusion injury. As reviewed in a previous section, BMAL1/CLOCK bipartite TF can modulate the diurnal oscillation of fatty acid oxidation, in part through transcriptional activity of a clock-output protein KLF15. REV-ERBα agonism protects against ischemic-reperfusion injury in the heart, though the detailed clock-controlled mechanism is not fully characterized (Stujanna et al., 2017). A transcriptional network including PER2-HIF1α and BMAL1/CLOCK-KLF15 is emerging as a clock-controlled checkpoint that licenses diurnal oscillation of cellular energy metabolism for metabolic reprogramming in ischemic heart disease (Figure 2).

#### Atherosclerosis

Atherosclerosis is a chronic process of plaque build-up in the vessel wall driven by lipid deposition and leukocyte infiltration to the subendothelial space (Wolf and Ley, 2019; Libby, 2021). The stenosis and restriction of the blood flow caused by the plaque make atherosclerosis the main cause of cardiovascular disease (Swirski and Nahrendorf, 2013). Epidemiological studies have demonstrated a strong connection between the disruption of circadian rhythms and atherogenic risk factors, such as lipid disorder and impaired glucose tolerance (Gooley, 2016; Poggiogalle et al., 2018). Leukocyte recruitment is greatly involved in the development of atherosclerosis (Swirski and Nahrendorf, 2013). In murine models of induced atherosclerosis using ApoE^–/–^ mice on a high-fat diet, neutrophils and monocytes were recruited to the atherosclerotic lesions rhythmically due to a morning peak of the CCL2 rhythm on the endothelium and the CCR2 rhythm on neutrophils and monocytes (Winter et al., 2018). Targeting the CCR2-CCL2 axis at a specific time achieved better attenuation of myeloid cell adhesion (Winter et al., 2018). Disturbing the rhythmicity of the SCN clock may be sufficient to promote atherosclerosis. For example, feeding low-fat diet to ApoE–/– mice generated more atherosclerotic lesions in aortic roots under constant light exposure, compared to feeding the same diet under normal light-dark cycles (Chalfant et al., 2020). Another mouse model using APOE^∗^3-Leiden mice with alternating light/dark cycles also exhibited more severe atherosclerosis with more macrophages in the lesion due to increased expression of ICAM-1 and CCL2 in the lesions (Schilperoort et al., 2020). The findings from these studies suggest that disturbance of the circadian rhythm can aggravate atherosclerosis. The rhythmic recruitment of myeloid cells to the atherosclerotic lesions driven by the circadian expression of the CCL2-CCR2 axis provides a mechanistic basis for chronotherapeutic treatments (Figure 4B).

### Neurodegenerative Diseases and Diabetic Neuropathy

Diabetic neuropathy is associated with a markedly reduced quality of life and poor prognosis (Callaghan et al., 2012). Impairment of cognitive function is increasingly being considered as an inevitable complication of type 2 diabetes mellitus (T2DM), and it has been associated with the risk of Alzheimer’s disease (AD) and vascular dementia. Considerable overlap has also been identified in the pathophysiology of T2DM and AD (Biessels and Despa, 2018). Sundown syndrome or sundowning, a well-known rhythmical neuropsychiatric phenomenon taking place in the late afternoon or early evening, is commonly observed in AD patients (Khachiyants et al., 2011). Another neuropsychiatric phenomenon is that pain intensity is often higher during night compared to day time. In addition, bright light exposure or exogenous melatonin supplementation has been found to improve sundowning symptoms, cognitive deficits, and sleep quality in AD patients (Roccaro and Smirni, 2020).

Emerging evidence supports the role of the circadian clock in the pathogenesis and progression of these neurological diseases. Pharmacological inhibition or genetic knockdown of REV-ERB was found to increase *Bmal1* transcription and to accelerate microglial uptake of amyloid-beta (Aβ) (Lee et al., 2020; Liu W. W. et al., 2020). Deletion of *Rev-Erbα* in an AD model was shown to decrease Aβ number and prevent the increase in AD-associated microglia markers such as TREM2 and CD45 (Lee et al., 2020). Exogenous light can inhibit the synthesis and secretion of melatonin, while the absence of light can promote production and secretion of melatonin. Melatonin can exhibit its effects regarding alleviating circadian disruption through regulation of related clock genes including PER1 and BMAL1 (Yamanaka et al., 2010), which have been confirmed to be associated with the development of neurodegenerative diseases. Deletion of *Per1/Per2* has been found to increase Chi3l1 expression in cerebrospinal fluid, which is a widely biomarker that is increased with the development of AD (Lananna et al., 2020). Loss of *Bmal1* in astrocytes was demonstrated to promote neuronal death due to aging and neurodegenerative diseases through the regulation of astrogliosis in a synergistic manner by a cell-autonomous mechanism and a lesser non-cell-autonomous signal from neurons (Lananna et al., 2018). These studies further confirmed that circadian clock-controlled checkpoints such as REV-ERBα play an important role in the pathogenesis of neurological diseases (Figure 5).

At present, accumulated β-amyloid (Aβ) in extracellular senile plaques and abnormally hyperphosphorylated tau in intracellular neurofibrillary tangles have been identified as the pathological features of AD (Polanco et al., 2018). Melatonin, produced by the pineal gland and associated with circadian rhythms, has been demonstrated to play an important role in the interaction with Aβ and tau pathology. Melatonin has been found to suppress the reduction of PI3K activity, pSer473 on Akt and pSer9 on GSK-3β, and this can lead to the reduction of Aβ aggregation in AD (Ali and Kim, 2015). Additionally, melatonin can effectively attenuate tau pathology in AD through regulation of kinases including death-associated protein kinase 1 (DAPK1) (Chen et al., 2020). In a post-transcriptional manner melatonin can significantly decrease DAPK1 expression in mouse primary cortical neurons and human neuronal cell lines. Moreover, melatonin directly binds to DAPK1 to increase DAPK1 degradation resulting in decreased tau phosphorylation at multiple sites related to AD (Chen et al., 2020). Based on these studies, it is clear that melatonin signaling is a promising clock-controlled checkpoint in the pathogenesis of AD and further exploration of effective therapeutic targets based on these findings is warranted.

Neuropathic pain is another important symptom in diabetic neuropathy, and this symptom can severely decrease the patients’ quality of life. One previous human study has suggested that patients’ pain intensity of diabetic neuropathy is often exacerbated at night (Gilron et al., 2013), indicating that pain intensity in diabetic neuropathy follows a circadian rhythm. However, the related mechanism has not yet been elucidated. One study attempted to characterize the circadian properties of neuropathic pain hypersensitivity in animal models of type I and type II diabetes (Akamine et al., 2018). Using a diabetic mouse model, they found that accumulation of sorbitol in the sciatic nerve may modulate the circadian properties of diabetes-induced neuropathic pain hypersensitivity. However, as described above, various forms of neuropathic pain share similar circadian properties. The special mechanism underlying the circadian rhythm of diabetes-induced neuropathic pain requires further research.

### Male Reproductive Disorder

The 24/7 modern lifestyle and social system has resulted in substantial changes in circadian behaviors of humans. Approximately 20 percent of the population are shift workers (Rajaratnam and Arendt, 2001). The social jetlag between work days and weekends is even more prevalent (Leypunskiy et al., 2018; Zhang et al., 2019). The effect of circadian disruption on the male reproductive system deserves attention. Several studies have analyzed the association between shift work and infertility risk, semen quality and/or reproductive hormones, as summarized by Caetano et al. (2020). Nevertheless, the results of these studies did not reach a consensus. One of the major reasons for this controversy may be that shift work consists of different work schedules, e.g., permanent night work and rotating shift work, which may lead to different health effects. A study of 1346 men in the community showed that rotating shift workers, but not permanent night workers, had lower sperm count compared to day workers (Liu K. et al., 2020). In the same publication, social jetlag between work days and free days was found to be quantitatively associated with decreased sperm count in 796 male university students.

Circadian disorder may contribute to the pathogenesis of male reproductive disorder in rodents. Male Sprague-Dawley rats with prolonged light exposure (20-h light:4 h-dark) were found to have increased sperm count and sperm motility and lower proportion of sperm abnormalities, while the rats with prolonged exposure to darkness (4-h light : 20-h dark) only showed a decreased proportion of sperm abnormalities (Moustafa, 2020). In contrast, another study reported that photoperiod changes only induced alterations in testosterone levels but had no effect on other characteristics such as semen quality and pregnancy rate after mating (Majrashi et al., 2017). Moreover, when pregnant rats were exposed to constant light, their male offspring showed a decrease in numerous indicators, including testosterone levels, number of Sertoli cells, sperm count and normal sperm count (Ogo et al., 2020). To simulate exposure to rotating shift work, a recent study put male mice into rotating light/dark cycles (Liu K. et al., 2020). The authors found that the sperm count of the male mice decreased, while the circadian oscillation of sperm count was not affected. In addition, sleep deprivation or sleep restriction is also hazardous to the male reproductive system (Chen et al., 2016). Despite numerous studies indicate the essential role of circadian disorder on the pathogenesis of male infertility, the underlying mechanism remains to be clarified (Figure 6).

In regards to the clock-controlled checkpoints in male reproductive system, several studies have suggested that reactive oxygen species (ROS) are activated in part through glutathione peroxidase, and superoxide dismutase (Torres et al., 2014; Alvarenga et al., 2015; Moustafa, 2020). Alteration of the testosterone levels were also observed in some circadian misalignment models (Qin et al., 2014; Arrebola and Abecia, 2017; Majrashi et al., 2017; Moustafa, 2020). The current knowledge around specific details of molecular events following the input of circadian misalignment is limited. Liu K. et al. (2020) suggested that the homologous recombination pathway was disrupted following circadian desynchrony in a rodent model. It remains unknown whether any of these molecular events are induced by unique signals. We speculate that BMAL1 may be a master regulator. BMAL1 is well known to govern the synthesis of testosterone (Alvarez et al., 2003), and regulates oxidative stress in other organ systems such as the vasculature and liver (Jacobi et al., 2015; Xie et al., 2020), although no such evidence is available for the testis. It is not clear whether disruption of the homologous recombination can also be induced by BMAL1, but the expression of core clock genes including BMAL1 is altered in the mouse testis (Liu K. et al., 2020). Twelve micro (mi)RNA are altered in the testes of Cry1 mutant mice, all of which are predicted to target the components of the core clock (Li C. et al., 2018). These findings deserve further confirmation under different exposure conditions. In addition, emerging evidence suggests that retinoic acid and its nuclear receptors are controlled by the clock during spermatogenesis (Bittman, 2016).

### Diabetic Complications Related to the Female Reproductive System

#### Gestational Diabetes Mellitus

Gestational diabetes mellitus (GDM) is a complication of pregnancy that has similar characteristics as type 2 diabetes mellitus (T2D), such as glucose intolerance, insulin resistance, and impaired insulin secretion (Catalano et al., 1999). The pathogenesis of GDM is associated with abnormal expression of circadian-related genes. In a cohort of 40 Greek pregnant women with GDM, four with T2D and 20 healthy pregnant women, significant reductions in the peripheral blood *BMAL1*, *PER3*, *PPARD*, and *CRY2* transcript levels were found in the GDM group, supporting the view that disorders of clock gene expression may play a pathogenic role in GDM (Ratajczak et al., 2010). Pappa et al. (2013a) studied *BMAL1* polymorphisms in GDM women and healthy controls and showed that the rs7950226 (G > A) and rs11022775 (T > C) polymorphisms of the *BMAL1* gene, combined with the rs7950226A/rs11022775C haplotypes were able to increase the susceptibility to GDM. In addition, the expression level of *BMAL1* mRNA from peripheral blood was significantly decreased in GDM patients compared to healthy controls (Pappa et al., 2013b).

Circadian neuroendocrine factors, such as glucocorticoids and melatonin, are altered in GDM (Pilorz et al., 2009; Sen and Hoffmann, 2020). Fabiś et al. (2002) found that melatonin increases blood insulin levels and decreases the synthesis of free fatty acids in experiments conducted on rats. In pancreatic β-cells, a genome-wide association analysis of 18,236 type 2 diabetic subjects demonstrated that mutations in melatonin receptor 2 (MT2) inhibits their response to melatonin, blocking the release of insulin from pancreatic β-cells (Prokopenko et al., 2009). Tuomi et al. (2016) also demonstrated that melatonin treatment reduced insulin secretion in risk G-allele carriers, which suggests that enhanced melatonin signaling decreases insulin secretion in the pancreas. More recently, genotyping of 1,025 Chinese women with a history of GDM showed that gestational weight gain may alter the effect of circadian-associated melatonin receptor 1B (MTNR1B) gene variants on long-term glucose changes (Nisa et al., 2018). GDM is known to be associated with chronic inflammation and the accumulation of oxidative damage in the placenta without affecting placental anatomy and their vascular structure in the majority of cases. Nevertheless, little has been studied on the distribution and pathophysiology of core clock genes in the GDM placenta.

#### Preeclampsia

Preeclampsia (PE) is characterized by hypertension and proteinuria after 20 weeks of gestation. The most accepted mechanism leading to the etiology of PE is shallow trophoblast invasion and abolished spiral artery remodeling that leads to placental hypoxia and oxidative stress. The most well-established link between circadian rhythm and PE is melatonin. Nakamura et al. (2001) reported that nocturnal melatonin levels in pregnant women with PE are significantly lower than those in normal pregnant women. Lanoix et al. (2012) also demonstrated reduced blood levels of melatonin in pregnant women with PE, compared to those with normal pregnancies. These results indicate that melatonin may be involved in the pathogenesis of PE. Melatonin is a potent antioxidant and may reduce the oxidative damage caused by ROS in the placenta (Manda et al., 2007; Tan et al., 2007; de la Sierra et al., 2009). Reiter et al. (2014) reported that melatonin protects cells against oxidative stress in the ovary and placenta. Another study has shown that melatonin prevents oxidative stress by inducing the expression and activity of catalase and superoxide dismutase, and inhibiting the expression of vascular endothelial growth factor (Valenzuela et al., 2015). In a mouse model, Lee et al. (2019a) found that melatonin reduces placental oxidative stress associated with intrauterine inflammation, which is capable of causing maternal placental malperfusion. A recent study demonstrated that the *CLOCK* gene may participate in the pathogenesis of PE via hypoxia. They found that the oscillation of *CLOCK* mRNA and protein levels are abnormal in the placenta of human patients and in rodent models of PE (Li Y. et al., 2020). The impairment of trophoblast proliferation, migration, and invasion under hypoxic conditions is able to be reversed by silencing the *CLOCK* gene in trophoblast cells (Li Y. et al., 2020). Therefore, studies on placental clock-controlled checkpoints in oxidative stress and the response to hypoxia may provide mechanistic insights into the pathogenesis of PE (Figure 7).

#### Preterm Birth

Preterm birth is defined as birth at a gestational age of fewer than 37 weeks and is one of the major causes of neonatal death worldwide (Kumar et al., 2017). Many studies have indicated that maternal shift work is related to preterm birth. In a study of occupationally exposed pregnancy cohorts, McDonald et al. (1988) found that a long work duration and shift changes were correlated with preterm birth. A cohort study of 845 female textile workers in China in 1992 showed that shift work increased the risk of preterm birth (Xu et al., 1994). Findings from a prospective cohort study of 1,908 pregnant women indicated that women who worked night shifts during pregnancy had a 50% increased risk of preterm delivery (Pompeii et al., 2005). Prospective cohort studies in the Nurses’ Health Study have suggested that night shift work is associated with an increased risk of early preterm birth (Whelan et al., 2007). In a study of 673 pregnant women from Singapore, they found that women with night-eating had a higher risk of preterm delivery and speculated that this may be due to the discrepancy between the timing of eating and circadian rhythms (Loy et al., 2020). Interestingly, one study used full-spectrum light at night to suppress maternal melatonin secretion which resulted in lower serum melatonin concentrations and fewer contractions in full-term pregnant women. The authors suggested that light therapy or melatonin therapy may have the ability to delay labor and overcome preterm birth (Olcese et al., 2013). Furthermore, Lee et al. (2019b) indicated that melatonin treatment could alleviate LPS-induced intrauterine/placenta inflammation and reduce preterm birth in mice by activating the silent information regulator transcript-1/nuclear factor-erythroid 2-related factor 2 signaling pathway. It is clear that further investigations should be conducted into the links between clock-dependent maternal inflammation in the placenta and placenta. However, meta-analyses studies showed that there is no association between shift work and preterm birth (Bonzini et al., 2011; van Melick et al., 2014). Shift work can dramatically alter sleep/wake rhythms and meal timing, which may also drive preterm birth. Therefore, studies eliminating these confounding factors should be conducted in order to unravel the underlying dysregulation of circadian oscillation caused by shift work and its implication for preterm birth.

### Obesity-Associated Circadian Regulation of Inter-Organ Communication

Obesity is a common comorbid risk factor in the pathogenesis of the complex disease, which is mechanistically linked to ectopic lipid deposition (Roden and Shulman, 2019) and metabolic-associated inflammation (metaflammation) (Hotamisligil, 2017). Mounting evidence supports the close links between circadian dysfunction and obesity, which can be referred to in previous reviews (Bass and Lazar, 2016; Panda, 2016; Reinke and Asher, 2019). Briefly, both irregular behavioral rhythms and clock dysfunction lead to obesity in rodents. Jet lag or constant light exposure contributes to leptin resistance (a hallmark of obesity), increased adiposity and weight gain (Shi S. Q. et al., 2013; Kettner et al., 2015). A high caloric diet increases food intake in the sleep phase, and results in a dampened daily rhythm of food intake (Kohsaka et al., 2007). Similarly, genetic mutation of core clock genes such as Bmal1, Clock, and Per2 leads to a dampened rhythm of food intake, and profound susceptibility to diet-induced obesity (Turek et al., 2005; Yang et al., 2009; Paschos et al., 2012).

Obesity is not merely a problem of overnutrition, but also a dysfunction of inter-organ communication, particularly in the adipocyte-brain axis. Adipocyte BMAL1 controls diurnal rhythms of de novo synthesis of polyunsaturated fatty acids (PUFA) through periodic expression of stearoyl-CoA desaturase 1 (SCD1) and long-chain fatty-acid elongase ELOVL6 (Paschos et al., 2012). The release of PUFA to the circulation may engage the hypothalamic circuit and inhibit food intake. However, the circadian rhythm of adipose SCD1 transcript is not found in major omics studies (source: CircaDB, CircaMetDB, CirGRDB). Instead, the SCD2 transcript oscillates robustly in adipose tissue. Adipocyte *O*-GlcNAc transferase promotes SCD1/2-controlled fatty acid desaturation and tissue accumulation of anandamide, which activates local cannabinoid receptor signaling and promotes diet-induced hyperphagia and obesity (Li M.-D. et al., 2018). Adipocyte *O*-GlcNAcylation may promote obesity through its well-established target clock proteins, such as BMAL1, CLOCK and PER2 (Durgan et al., 2011; Kim et al., 2012; Kaasik et al., 2013; Li et al., 2013, 2019; Liu et al., 2021). Adipocyte REV-ERBα limits adipocyte expansion under a high caloric diet (Hunter et al., 2021). Deletion of *Rev-Erbα* in adipocytes results in profound obesity without adipose inflammation and fibrosis. It appears that REV-ERBα controls SCD1 expression and fatty acid desaturation in adipose tissue (Hunter et al., 2021).

Despite these findings, the receiving end in the brain is not fully characterized. Recently, Cedernaes et al. (2019) have shown that clocks in AgRP neurons (hunger neurons) of hypothalamus govern circadian transcriptional response to leptin, a critical adipose-secreted endocrine factor (Cedernaes et al., 2019). AgRP neurons project to the paraventricular nucleus (PVN) of hypothalamus, which coordinates neuronal inputs to elicit feeding and satiety. Kim E. R. et al. (2020) reported that the PVN-clock determines the diurnal rhythm of energy metabolism through rhythmic sensitivity to GABAergic inputs. Ablation of the PVN-clock results in obesity. Thus, clock-controlled checkpoints involved in the metabolism and signaling of PUFA, anandamide, and leptin may coordinately link adipocytes and hypothalamic neurons, which maintain energy homeostasis through inter-organ communication.

The hypothalamic-pituitary-adrenal (HPA) axis provides another potent means to mediate inter-organ crosstalk during obesity. Cushing syndrome presents extreme obesity when HPA goes wrong. Both hyper-cortisolism and low-amplitude rhythm of plasma glucocorticoids are well-known indicators of natural aging from rodents to nonhuman primates and humans (Thompson et al., 2020). However, it is still unsolved whether and how the circadian rhythm of HPA is involved in the pathogenesis of the complex disease. Adrenal gland-specific manipulation of the core clock genes would provide insights into this question.

In addition, obesity-associated inter-organ communication can take place between peripheral tissues. Metabolite profiling of diet-induced obese mice reveals that obesity decreases the temporal correlation of metabolites among seven organs and serum (Dyar et al., 2018b). Comparing metabolites in alanine metabolism and gluconeogenesis among organs and serum suggests that the glucose-alanine cycle, which transports amino acids from the muscle to liver for glucose production, is highly active in obesity (Dyar et al., 2018b). Though still in the early stage, the application of multi-omics, and single-cell omics would facilitate the elucidation of clock-controlled checkpoints in the obesity-associated inter-organ communication, which holds promise for diagnosis, prevention, and treatment of complex disease.

## Time Medicine

As we reviewed above, the circadian clock is widely involved in the pathophysiology of complex diseases, particularly metabolic diseases. Time medicine is emerging as an interdisciplinary field to understand and target clock-controlled checkpoints for the improvement of well-being and health (Cederroth et al., 2019; Ruben et al., 2019; Allada and Bass, 2021; López-Otín and Kroemer, 2021). Time medicine includes two major parts, namely, clinical treatment based on the internal time, and drugging the clock.

### Clinical Treatment Based on the Internal Time

For optimizing the time of clinical treatment, the time-of-day effect of both drug treatments and surgeries needs to be extensively determined in future clinical studies. Proof-of-principle examples are bedtime dosing of the anti-dyslipidemia drug statins and afternoon heart surgeries (Cederroth et al., 2019). It is reported that more than 75% of drugs from 106 clinical trials have shown obvious time-of-day effects or toxicity (Ruben et al., 2019), corroborating the idea that circadian time should be taken into consideration at both the pre-clinical and clinical stages (Cederroth et al., 2019). The onsets of complex diseases show a diurnal pattern. For example, cancer pain erupts at 10 a.m., stroke and sudden cardiac death usually occurs between 6 and 12 a.m., and asthma occurs at 4 a.m. Therefore, detailed clinical logs are also required for time-optimization of clinical treatment.

While hormones such as melatonin and glucocorticoids are well-established biomarkers of the circadian rhythm, tissue-specific biomarkers are largely unexplored. The circadian transcriptome is usually the starting point to identify tissue-specific biomarkers for chronotherapy. However, it is impractical to obtain clinical samples (especially for the brain) suitable for circadian studies, which normally require frequent sampling around the clock with decent temporal resolution. To fill the gap, an unsupervised algorithm called cyclic ordering by periodic structure (CYCLOPS) was developed to order clinical samples into circadian structure without time indication (Anafi et al., 2017). CYCLOPS analysis of RNA-seq data in 13 human tissues indicates that nearly half of the protein coding transcriptome is rhythmic in at least one tissue (Ruben et al., 2018). Most excitingly, they found that nearly a thousand of these cycling genes, which are involved in drug delivery and metabolism, or as drug targets, may mediate time-of-day drug efficacy. Empirical validation of these findings and large-scale input of data into CYCLOPS would improve the precision and accuracy of circadian biomarkers.

Prediction of the circadian phase in vivo is another important aspect for optimizing the time of clinical treatment. Due to the dynamic nature, profiling the circadian transcriptome atlas of human tissues is not enough but the phase information by itself is equally important for optimization of the time of clinical treatment. The prediction of the circadian phase of an individual’s drug target tissue(s) is a hot topic. In order to achieve this, several algorithms were invented, including Molecular-Timetable, ZeitZeiger, BIO-CLOCK, PLSR, and Time-Signature (Naef and Talamanca, 2020). The phase prediction process mainly includes four steps: training algorithms with time-indicated RNA seq data to extract biomarkers, building low-dimensional circadian trajectory, cross validation with known time labeled sample, and finally inferring the unknown sample’s phase. Using dim light melatonin onset (DLMO) as an SCN phase indicator, the accuracy of these algorithms was verified through inferring the phase of SCN, with a maximum prediction error of approximately 3 h. Besides SCN, more druggable tissues’ specific biomarkers and clinical feasible phase prediction methods need to be developed in the future.

### Drugging the Clock

Dysregulation of the circadian rhythm is a hallmark of complex diseases (López-Otín and Kroemer, 2021). Alteration of the period length results in abnormal sleep-wake patterns (Ashbrook et al., 2020). Circadian amplitude damping always precedes neurodegenerative disorders and accelerates aging related disorders (Abbott et al., 2020). The promising efficacy of time-restricted feeding in preclinical anti-obesity and anti-cardiometabolic disease studies indicates a robust phase misalignment in these complex diseases related to life style (Panda, 2016). The scheduling of light exposure and diet quality represents a handy approach to restore circadian rhythms and health. For example, short-term exposure to bright light shifts the phase of SCN and alleviates jet-lag or circadian related mood disorders (Blume et al., 2019). Time-restricted eating improves the metabolic profile in cardiometabolic diseases (Panda, 2016). Future work targeting clock-controlled checkpoints hold great promise for translating these mechanisms into clinical practice and devising small chemicals for applications in people that have compliance issues with these cues.

Clock-modulating compounds represent a highly valuable approach to reset the clock. Several small molecules have been identified from chemical screening (Ruan et al., 2021). GSK3-β and CKIε/δ inhibitors were discovered from the LOPAC library (Library of Pharmacologically Active Compounds) to shorten or lengthen the circadian period. CRY1 stabilizer KL001 increases the circadian period and inhibits hepatic glucose production in vitro. SR9009/SR9011 and Nobiletin regulate circadian amplitude through REV-ERB/ROR agonism (He et al., 2016; Nohara et al., 2019). Notably, cordycepin shifts circadian phase in both central and peripheral clocks through RUVBL2-mediated circadian chromatin remodeling (Ju et al., 2020). Structure-based virtual screen is an alternative method, which is time and labor saving. Encouragingly, a recent virtual screen based on the crystal structure of melatonin receptor 1 uncovered several bioactive molecules from 150 million ‘lead-like’ molecules, which may have potentially therapeutics significance (Stein et al., 2020). Another study using a molecular-docking approach also found small molecules which have circadian amplitude-modulating activity, binding to CLOCK and disrupting its interaction with BMAL1 (Doruk et al., 2020). In light of these advances, more clock-modulating compounds targeting clock-controlled checkpoints would likely be available in the near future and their clinical efficacy would be tested for better care and treatment of complex diseases.

## Conclusion

Over the past two decades, circadian regulation of physiology and metabolism has been the key direction of circadian rhythm research. Substantial progress has been made in elucidating circadian clock-controlled pathways and checkpoints in peripheral tissues and their relevance in complex diseases. Despite these achievements, the majority of the research has been focused on a few prototypical organs, such as the liver, and to a lesser degree, the heart and muscle. We anticipate that the next phase of this research would lead to translational medicine in liver disease, deep mechanistic insights in circadian biology and medicine related to the heart and muscle, and further findings in less characterized tissues and organs relevant to complex diseases.

## Author Contributions

M-DL: conceptualization, investigation (intro and liver), and supervision. HX: investigation (liver) and visualization. YY and WH: investigation (blood). XY and GD: investigation (neuron). HL, HZ, and T-LH: investigation (female reproduction). DT, FD and ZZ: investigation (heart). T-LH: investigation (female reproduction). QC: investigation (male reproduction). DJ: investigation (intro and time medicine). KC: investigation (muscle). M-DL, HX, YY, XY, HL, DT, HZ, ZZ, T-LH, QC, GD, DJ, KC, FD, and WH: writing. All authors contributed to the article and approved the submitted version.

## Conflict of Interest

The authors declare that the research was conducted in the absence of any commercial or financial relationships that could be construed as a potential conflict of interest.

## Publisher’s Note

All claims expressed in this article are solely those of the authors and do not necessarily represent those of their affiliated organizations, or those of the publisher, the editors and the reviewers. Any product that may be evaluated in this article, or claim that may be made by its manufacturer, is not guaranteed or endorsed by the publisher.
